# Incidence of anogenital warts in Germany: a population-based cohort study

**DOI:** 10.1186/1471-2334-10-360

**Published:** 2010-12-23

**Authors:** Angela A Kraut, Tania Schink, Renate Schulze-Rath, Rafael T Mikolajczyk, Edeltraut Garbe

**Affiliations:** 1 Department of Clinical Epidemiology, Bremen Institute for Prevention Research and Social Medicine (BIPS), Achterstrasse 30, 28359 Bremen, Germany; 2 Sanofi-Pasteur MSD GmbH, Paul-Ehrlich-Straße 1, 69181 Leimen, Germany

## Abstract

**Background:**

Human papilloma virus (HPV) types 6 and 11 account for 90 percent of anogenital warts (AGW). Assessment of a potential reduction of the incidence of AGW following introduction of HPV vaccines requires population-based incidence rates. The aim of this study was to estimate incidence rates of AGW in Germany, stratified by age, sex, and region. Additionally, the medical practitioner (gynaecologist, dermatologist, urologist etc.) who made the initial diagnosis of AGW was assessed.

**Methods:**

Retrospective cohort study in a population aged 10 to 79 years in a population-based healthcare insurance database. The database included more than 14 million insurance members from all over Germany during the years 2004-2006. A case of AGW was considered incident if a disease-free period of twelve months preceded the diagnosis. To assess regional variation, analyses were performed by federal state.

**Results:**

The estimated incidence rate was 169.5/100,000 person-years for the German population aged 10 to 79 years. Most cases occurred in the 15 to 40 years age group. The incidence rate was higher and showed a peak at younger ages in females than in males. The highest incidence rates for both sexes were observed in the city-states Berlin, Hamburg and Bremen. In females, initial diagnosis of AGW was most frequently made by a gynaecologist (71.7%), whereas in males, AGW were most frequently diagnosed by a dermatologist (44.8%) or urologist (25.1%).

**Conclusions:**

Incidence of AGW in Germany is comparable with findings for other countries. As expected, most cases occurred in the younger age groups. The frequency of diagnoses of AGW differs between sexes and women and men receive treatment by doctors of different specialties.

## Background

Anogenital warts (AGW) are a clinical manifestation of human papillomavirus (HPV) infection. More than 100 HPV types have been recognised so far, of which more than 40 can infect the anogenital tract [1,2]. Genital HPV is a very common sexually transmitted infection [3]. Some as high risk considered HPV types are associated with the vast majority of cervical and other anogenital cancers [4,5]. HPV type 6 and in a smaller proportion type 11 account for 90 percent of AGW [4-6]. A tetravalent vaccine against HPV 6, 11, 16 and 18 which protects against AGW and other manifestations of HPV infection has been approved by the European Medicines Agency (EMA) in 2006 [7].

While AGW can resolve spontaneously, remain unchanged or worsen when left untreated, often treatment is chosen by the patients incurring treatment costs [8]. Incidence rates of AGW have been reported for several countries, showing a wide range of estimates [9-18], even in studies originating from the same country [9,10]. Differences in reported incidence rates can be due to several factors, including the use of different data sources, study methods, and epidemiology of HPV between or within countries. Studies reported differences between sexes [9-11,13-17], unless the study population was restricted to one sex, e.g. women [12,18,19].

A recent study estimated the annual incidence of AGW in Germany as 171 per 100,000 women aged 14 to 25 years [12]. However, since the study was based on a relatively small convenience sample of physicians, population-based data on AGW are lacking for Germany. We therefore used a health insurance claims database including records on more than 14 million persons to estimate incidence rates of AGW in a population-based framework in Germany. Furthermore, we investigated the specialty of the medical practitioner who diagnosed AGW.

## Methods

### Study Design and Data Source

The study was conducted in a retrospective cohort design. Source of data was the German Pharmacoepidemiological Research Database (GePaRD) which has been described elsewhere [20]. The GePaRD is a database of more than 14 million insurees of all ages (approximately 17% of the German population) and covers all geographical regions of Germany. The database consists of records of four large statutory health insurance companies (SHI). The data is not publicly available but other institutions could apply for the data of the respecitve SHIs and years. Statutory health insurance is mandatory in Germany for all persons below a certain income threshold. Above this threshold persons are permitted to choose private insurance companies instead. However, given the relatively high threshold and the fact, that some persons with a high income still elect statutory health insurance, about 90% of the German population remain in statutory health insurance. When this project started, the database contained data for the years 2004-2006 for the different insurance companies. Since then, the database has been extended to include data from more recent years.

The GePaRD contains demographic information, information on hospital admissions, ambulatory physician visits and prescriptions. The hospital data contains information on admission and discharge dates and on diagnostic and therapeutic procedures carried out in hospital with the respective date. Ambulatory physician visit claims data include ambulatory treatments, procedures, and diagnoses. Since ambulatory physician visits are reimbursed quarterly, ambulatory diagnoses can only be allocated to a quarter of the year. All diagnoses, inpatient and ambulatory diagnoses, are coded according to the German Modification of the International Classification of Diseases, 10^th ^Revision (ICD-10-GM) [21]. Preliminary analyses regarding the age and sex distribution, the number of hospital admissions, and drug use demonstrated that the database is generally representative of information published in official statistics [20]. Since the proportion of the population included in the database varies across German federal states, regional weights are necessary to obtain an estimate representative for the whole of Germany. The utilisation of health insurance data for scientific research is regulated by the Code of Social Law in Germany (SGB X). Approval for the use of the data used in this study was granted by all SHIs that contributed data to the study and the Federal Ministry of Health. A detailed data protection concept was approved by the Federal Ministry of Health which precludes the use of data outside of the Bremen Institute for Prevention Research and Social Medicine. Informed consent was not required by law, since the study was based on pseudonymous data.

### Definitions

#### Case identification

Diagnoses of AGW were ascertained by using the ICD-10-GM (version valid in 2006) code A63.0, a specific code for anogenital (venereal) warts. In order to estimate incidence rates, we only considered newly diagnosed cases for this analysis. Each insuree had to have an AGW free period of 12 months preceding the diagnosis. Cases were only considered from 2005 onwards, since the year 2004 served to exclude prevalent cases of AGW. In case of several diagnoses of AGW in the same patient in different quarters, these were counted as a single episode of AGW when the time interval between two diagnoses of AGW was less than 12 months. Since ambulatory data are only available by quarter, the date of the ambulatory diagnosis was set to be at the middle of each quarter. Some insurees could have had more than one episode, thus persons could be counted as incident cases more than once.

#### Cohort entry and exit

Cohort entry was on 1st of January 2005 or on the first date afterwards, when patients had been continuously insured 12 months preceding cohort entry and not had a diagnosis of anogenital warts during this time period. Cohort members had to be between 10 and 79 years of age at cohort entry and remained in the cohort until the first of the following dates: reaching of age 80, the day of the first diagnosis of AGW, end or interruption of insurance, death or end of the study period (31.12.2006).

#### Regional analysis

To investigate regional differences, we estimated incidence rates by federal state. A specific characteristic of the German federal states is that three major cities (Berlin, Hamburg and Bremen) are federal states themselves, so called city-states. These three city-states present densely populated areas and can serve as a proxy for urban regions. For the purpose of description of regional differences regarding predominantly rural and urban regions on the one side and west-east comparison on the other side, federal states were classified into three groups: city-states, other former West-German federal states, and other former East-German federal states.

#### Specialty of medical practitioners

For the descriptive analysis of the specialty of the medical practitioner who initially diagnosed AGW, only the first episode for each patient was considered. The specialty of the medical practitioner was only available for doctors not practicing in group practices. In the coding system, general practitioners can not be distinguished from group practices and therefore built a common category. The specialties of medical practitioners included in the analysis were gynaecologists, dermatologists, urologists, general practitioners/group practices, surgeons, and internists. As no exact date, but only the quarter was known for ambulatory diagnoses, it was not possible to establish which medical practitioner diagnosed anogenital warts first in cases where more than one ambulatory diagnosis was made in the same quarter. In these cases, the specialty of the medical practitioner was classified as "not identifiable".

### Statistical analysis

#### Estimation of incidence rates

The crude incidence rate and incidence rates stratified by age, sex and region were calculated for the time period 2005 to 2006. The number of incident cases of the respective stratum was divided by the total person time of the respective stratum, expressed per 100,000 person-years (py). Confidence intervals for incidence rates were obtained from the Poisson distribution [22]. For comparison purposes with incidence rates reported in the literature, incidence rates for the age group 14 to 65 years were calculated.

Incidence estimates were validated by independent programming by a second programmer and all other analyses by independent code review. All statistical analyses were conducted using SAS statistical software version 8.2.

#### Standardisation of incidence rates

The incidence was stratified by sex, five year age groups and the 16 federal states. An estimate of incidence for Germany was obtained using population weights taking age (14 categories), sex (2 categories) and federal state (16 categories) into account. For each of the resulting 448 strata, population weights were obtained for the German population in 2006 based on data from the German Federal Statistical Office [23]. In order to account for differences in regional age structure, incidence rates for federal states were directly standardised for the German population aged 10 to 79 years in 2006.

#### Assessment of the specialty of the medical practitioner

For the assessment of the specialty of the medical practitioner who initially diagnosed AGW, only the first diagnosis of AGW during the time in the cohort was considered for each insuree.

#### Geographical distribution of incidence rates

The graphical presentation of the incidence rates in a geographical map was conducted with ESRI^® ^ArcMap™, version 9.3. The classification of incidence rates for this geographical mapping was performed using natural breaks, where classes are based on natural groupings inherent in the data and not on equal intervals. This grouping method is based on the ArcGIS implementation of the Fisher-Jenks algorithm and minimizes differences between data values within classes and maximises differences between classes [24,25].

## Results

### Incidence rates

In total, there were 11,206,905 insurees in the age group 10-79 in our cohort, who contributed 20,686,223 py. There were 35,422 incident diagnoses of AGW, accounting to an annual crude incidence of 171.2 per 100,000 py in the studied period.

The overall weighted estimate for the 10 to 79 year old population of Germany was very similar (169.5 per 100,000 py) and was 1.29 times higher for women than for men (191.10 versus 147.66 per 100,000 py respectively).

**Figure 1:**
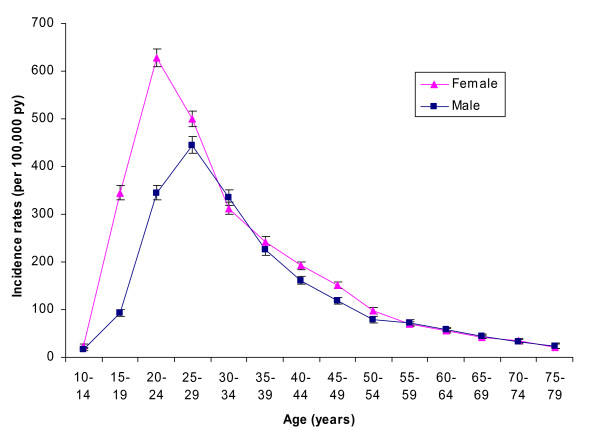
**Incidence rates of anogenital warts by 5 year age groups in males and females for Germany in 2005 to 2006**.

The incidence rates were very similar in 2005 and 2006 (Table 1). When the incidence was further stratified by age, females had higher incidence rates of AGW than males in the younger age groups, with very little difference after the age of 30 years (Figure 1).

**Table 1:** Crude incidence rates of anogenital warts by age and sex

Year	Age group (years)	Sex	Person-time*	Cases	Incidence rates**	95% Confidence interval**
**2005**	**10-79**	Female	56.85	10,999	193.47	189.87 to 197.12

		Male	45.74	6,613	144.57	141.10 to 148.09

		Total	102.59	17,612	171.67	169.14 to 174.22

	**14-65**	Female	46.43	10,676	229.93	225.59 to 234.33

		Male	36.93	6,339	171.64	167.44 to 175.92

		Total	83.36	17,015	204.11	201.05 to 207.20

**2006**	**10-79**	Female	57.59	10,901	189.30	185.76 to 192.89

		Male	46.69	6,907	147.95	144.48 to 151.48

		Total	104.27	17,808	170.78	168.29 to 173.31

	**14-65**	Female	46.60	10,586	227.19	222.88 to 231.56

		Male	37.34	6,645	177.97	173.72 to 182.31

		Total	83.93	17,231	205.30	202.24 to 208.39

* in 100,000 person-years

** per 100,000 person-years

The peak incidence was higher in females (627 per 100,000 py; 95% CI 601 to 654) than in males (457 per 100,000 py; 95% CI 433 to 482), and occurred at an earlier age in females (20-24 years) than in males (25-29 years).

**Figure 2:**
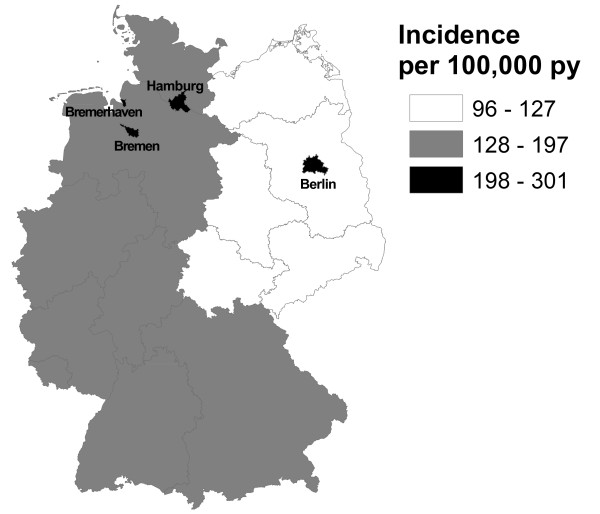
**Geographical variation in incidence rates of anogenital warts in Germany 2005 to 2006**. Note: Incidence rates are standardised for the sex and age distribution of the German population in 2006 and are computed per 100,000 person-years. Bremen and Bremerhaven constitute the city-state Bremen.

When the same age and sex population structure was applied across all federal states, the differences persisted between city-states and other regions on the one side and between former East and West Federal States on the other side (Figure 2).

**Figure 3:**
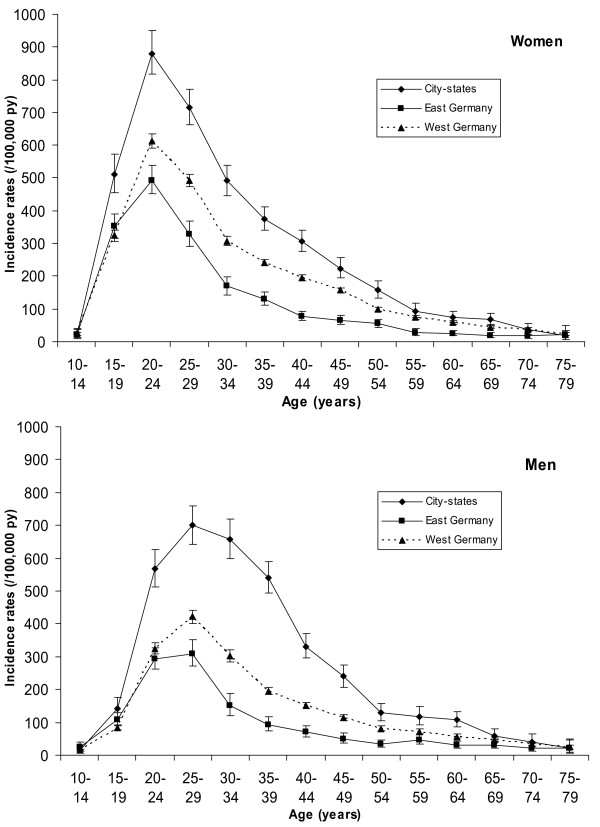
**Crude incidence rates of anogenital warts for regions in Germany in 2005 to 2006 by sex**. City-states: the Federal states Berlin, Bremen and Hamburg. West Germany: Federal states belonging to former West Germany without city-states. East Germany: Federal states belonging to former East Germany without city-states.

When Germany was stratified into city-states (Berlin, Hamburg, Bremen), former East Germany and former West Germany (Figure 3), another feature of the incidence distribution became apparent: In the city-states, a high incidence among men extended over a wider age range, so that in the age group 30-39 years the incidence was higher among men than among women. After the age of 40 years sex differences in AGW incidence disappeared also in the city-states.

### Specialty of medical practitioner who made the first diagnosis of AGW

**Figure 4:**
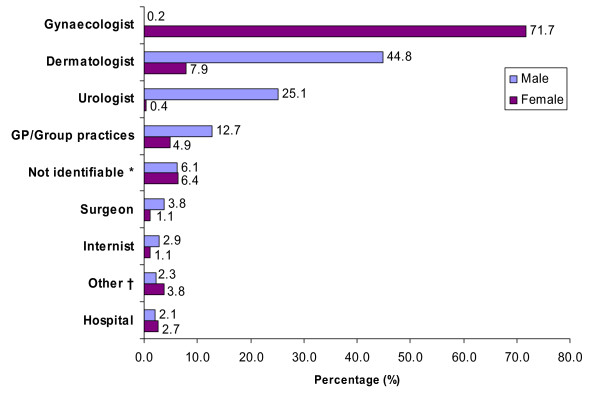
**Initial diagnosis of anogenital warts by specialty of medical practitioner (2005 to 2006)**. Note: hospital diagnoses are not classified by specialties. * The medical practitioner who made the first diagnosis could not be ascertained because more than one medical practitioner made a diagnosis in the respective quarter. † Ambulatory diagnoses made by medical practitioners with other specialties than those listed.

Women were mostly diagnosed by gynaecologists whilst men were most frequently diagnosed by dermatologists, followed by urologists (Figure 4).

## Discussion

Previous studies estimated the incidence of AGW for Australia [13,19,26], Canada [14,16], United States [15,27], UK [9,10], Italy [18], Spain [19], France [28], and Germany [12]. While due to the use of different data sources, study methods and reporting of the incidence for various age ranges a direct comparison is difficult, some observations can be made. The overall incidence (for all ages) was highest (219 per 100,000) in Australia [26], with most other estimates around 150 per 100,000, matching well the estimates from the current study. Another German study using a convenience sample of gynaecologists found an incidence of 76 per 100,000 in Germany in women of all ages and 114 per 100,000 in women of the age group 14-65 [12]. Both estimates were about 50% lower than estimates from the current study using a systematic approach to obtain population based data. Several studies reported also incidence among females in the age group 20-24 years: with 861 per 100,000 the incidence was highest in Australia [17], followed by UK (672 per 100,000) [9] - both slightly higher than the estimates from the current study (627 per 100,000 from Figure 1). Substantially lower rates were reported from Canada, 466 per 100, 000 in Manitoba [14] and 338 in British Colombia [16] in line with the lower overall incidence of genital warts in Canada.

The peak incidence at a younger age in women than in men observed in our study is consistent with findings from several previous studies [16,17,27]. The overall sex differences in incidence rates of AGW reported in the literature vary. While Castellsagué et al.[11] reported higher incidence rates in men than in women (136.58 per 100,000 in men and 99.95 per 100,000 in women) for the 14 to 64 year old population of Spain, Marra et al.[16] reported higher overall incidence rates in men than in women (131 per 100,000 in men and 121 per 100,000 in women in 2006), but also higher age specific incidence rates for women than for men in the age group 15 to 25 years. A Canadian study by Kliewer et al. reported age standardised incidence rates over time and showed a reversion of the sex ratio after the year 2000 from higher incidence rates in women to higher incidence rates in men [14].

Our study clearly demonstrated different pathways to diagnosis of AGW for male and female patients. This question was not assessed in previous studies, possibly because of the methodological restrictions: some studies were conducted with data from primary care or specialised clinics [9,10], included only one or selected specialties of medical practitioners for case ascertainment [11,12,18] or were population-based but did just not investigate the specialty of the medical practitioner who diagnosed AGW first [14,16]. However, another reason can be that health care systems vary across countries and that the sex difference observed for the diagnosing medical practitioner is mainly associated with the organisation of the German health care system which enables a direct access to specialists located in private practices without consulting the general practitioner first. These different pathways can also partly explain the higher incidence noted in younger women than in men: in women, AGW can be chance findings during regular visits to the gynaecologist, while men have to seek treatment actively.

Clear differences in incidence of AGW were found between the territorial states of former East and West Germany, and the city-states. The higher incidence in city-states was not surprising as these regions were used as proxies of urban regions and sexually transmitted diseases are often more common in urban settings. The difference between former East and West Germany is possibly also related to a higher level of urbanisation in former West Germany, but might also be an expression of many differences persisting between both former parts of Germany.

The comparison of just two years did not demonstrate any difference in AGW incidence - other studies which compared longer time periods typically demonstrated an increase in the incidence over time [14-16]. A decrease in the incidence of AGW can be expected in Germany starting from 2007 as in October 2006, the quadrivalent HPV vaccine Gardasil^® ^which protects from HPV types 6 and 11 was licensed for European countries including Germany [29] and is recommended since July 2007 for girls of 12-17 years of age [30].

### Strengths and Limitations

The main strength of this study is the population based nature of the data source which has been shown to be adequately representative for Germany with respect to age and sex [19]. The large sample of routinely collected data diminishes potential selection bias which may result from identification of patients in private practices, hospitals or from clinics specialised in diagnosis and treatment of sexually transmitted diseases, particularly if the identification of patients was based on a convenience sample of private practices or hospitals [8,10,11]. The size of the data source also allowed estimation of incidence rates in very narrow age strata for men and for women and with high precision. In contrast to GP-based studies [9,12,16], our study included patients diagnosed by all specialties of medical practitioners.

There are also some limitations. Patients of higher socioeconomic status could have been underrepresented due to their option to join private health insurances, which includes about 10% of the German population. Miscoding and the use of unspecific codes by health care professions such as "viral warts" might have happened and would have led to an underestimation of the incidence in our study. Due to the diagnosis based nature of the case ascertainment, only patients who went to see a medical practitioner and who were diagnosed with AGW could be included.

## Conclusions

We conducted an observational database study to estimate incidence rates of AGW in Germany in a population-based framework. Incidence of AGW was found in the range of findings for other countries and was substantially higher than in a previous German study using a convenience sample. High incidence rates were concentrated in the younger age groups, whereas considerable incidence rates were also noted above the age of 34 years in both, men and women. A reduction in the incidence of AGW may be achievable through a vaccination programme using a vaccine targeting the HPV types 6 and 11. Further research is recommended.

## Competing interests

Funding for this study was provided by Sanofi Pasteur MSD GmbH, Leimen, Germany. RS is employed by the sponsor company.

## Authors' contributions

All authors were involved in the study conception, study design, and interpretation and validation of the results. AAK, RTM and EG were involved in drafting the manuscript, TS conceptualised and performed the statistical analysis. TS and RS have critically revised the final manuscript. All authors read and approved the final manuscript.

## Pre-publication history

The pre-publication history for this paper can be accessed here:

http://www.biomedcentral.com/1471-2334/10/360/prepub
